# Comparative analysis of the gonadal transcriptomes of the all-female species *Poecilia formosa* and its maternal ancestor *Poecilia mexicana*

**DOI:** 10.1186/1756-0500-7-249

**Published:** 2014-04-17

**Authors:** Ina Maria Schedina, Stefanie Hartmann, Detlef Groth, Ingo Schlupp, Ralph Tiedemann

**Affiliations:** 1 Unit of Evolutionary Biology/Systematic Zoology, Institute of Biochemistry and Biology, University of Potsdam, Karl-Liebknecht-Straße 24-25, Haus 26, 14476 Potsdam, Germany; 2 Department of Bioinformatics, Institute of Biochemistry and Biology, University of Potsdam, Karl-Liebknecht-Straße 24-25, Haus 14, 14476 Potsdam, Germany; 3 Department of Biology, University of Oklahoma, 730 Van Vleet Oval, Norman, OK 73019, USA

## Abstract

**Background:**

The Amazon molly, *Poecilia formosa* (Teleostei: Poeciliinae) is an unisexual, all-female species. It evolved through the hybridisation of two closely related sexual species and exhibits clonal reproduction by sperm dependent parthenogenesis (or gynogenesis) where the sperm of a parental species is only used to activate embryogenesis of the apomictic, diploid eggs but does not contribute genetic material to the offspring.

Here we provide and describe the first *de novo* assembled transcriptome of the Amazon molly in comparison with its maternal ancestor, the Atlantic molly *Poecilia mexicana*. The transcriptome data were produced through sequencing of single end libraries (100 bp) with the Illumina sequencing technique.

**Results:**

83,504,382 reads for the Amazon molly and 81,625,840 for the Atlantic molly were assembled into 127,283 and 78,961 contigs for the Amazon molly and the Atlantic molly, respectively. 63% resp. 57% of the contigs could be annotated with gene ontology terms after sequence similarity comparisons. Furthermore, we were able to identify genes normally involved in reproduction and especially in meiosis also in the transcriptome dataset of the apomictic reproducing Amazon molly.

**Conclusions:**

We assembled and annotated the transcriptome of a non-model organism, the Amazon molly, without a reference genome (*de novo*). The obtained dataset is a fundamental resource for future research in functional and expression analysis. Also, the presence of 30 meiosis-specific genes within a species where no meiosis is known to take place is remarkable and raises new questions for future research.

## Background

The evolution as well as the costs and benefits of sexual reproduction are central topics in evolutionary biology [1]. Despite theoretically well-defined costs of sexual reproduction, most prominently the twofold cost of producing males [2,3] that leads to a lower population growth rate for any sexual species, only about 0.1% of animal species have an asexual reproduction strategy [4]. Why this distribution prevails is an important puzzle in evolutionary biology. One consequence of asexual reproduction is the absence of full recombination. This probably impedes adaptation to changing environmental conditions and can lead in the long term to the accumulation of negative mutations, a phenomenon known as Muller’s Ratchet [5].

An especially interesting mode of reproduction is gynogenesis or sperm-dependent parthenogenesis [6,7]. Gynogenetic species are unisexual and therefore reproduce clonally, but they need sperm of males of closely related species to initiate embryogenesis. Usually, there is no contribution of the paternal genome to the next generation. Gynogenesis hence combines disadvantages of sexual and asexual reproduction.

Gynogenetic species typically originate through hybridisation of two sexually reproducing species [8] and can be thus considered as “frozen F1’s” [9], as no recombination occurs in subsequent generations after the hybridisation event.

An example for an asexual/sexual species complex is the gynogenetic Amazon molly, *Poecilia formosa* (Teleostei: Poeciliinae) and its parental species. The Amazon molly was the first unisexual vertebrate to be described [10]. It evolved presumably at least 120,000 years ago through a single hybridisation event [11] between a female Atlantic molly, *P. mexicana*[12], and a male sailfin molly, *P. latipinna*[13]. So far all experiments to artificially create Amazon molly like unisexuals through hybridisation of the parental species did not succeed [10,14,15], but always yield sexual F_1_ hybrids.

Amazon mollies occur in sympatry with at least one host species along the coastal versant of northern Mexico [16-18]. The Amazon molly produces diploid eggs without meiosis [19]. Embryogenesis is triggered by sperm of closely related males of three species, both parental species, *P. latipinna* and *P. mexicana*[10,17], and the Tamesí molly, *P. latipunctata*[20,21]. This pseudo-fertilization is internal and males of the sperm donor species have to copulate with the females of the Amazon molly for insemination, which has been described as a parasitic relationship of the Amazon molly with its sperm donor species [7]. Except in the rare case of paternal introgression where the complete [22] or parts of (microchromosomes [23]) the genetic material of the paternal species are passed on to the next generation, the reproduction of the Amazon molly is strictly clonal. There is no meiosis during the development of the gametes, such that new genetic variation only originates from mutations [19].

The Amazon molly is an excellent model for investigating the mechanisms of parthenogenetic, in particular gynogenetic reproduction, especially in comparison with its bisexual ancestors, as all these species are biologically very similar and mainly differ in their mode of reproduction (gynogenetic vs. bisexual) [7].

In the present study we compared the gonadal transcriptome of the Amazon molly, *P. formosa* and its maternal ancestor, the Atlantic molly, *P. mexicana*. Transcriptomic data were produced by Illumina sequencing and the obtained dataset will contribute significantly to unravel the evolution and mechanisms of the gynogenetic reproduction mode.

Furthermore we identified genes relevant for recombination and meiosis through comparative analysis. These genes will be functionally characterized in the future, in order to better understand mechanisms of gynogenesis and the genomic consequences of sexual vs. asexual reproduction.

## Methods

### Library construction and transcriptome sequencing

Samples were taken for each species (*P. formosa* and *P. mexicana*) from two laboratory born, fully mature females of same age and constitution (kept at the University of Potsdam). The founder fishes of the *P. mexicana* stock were collected in 1994 from the Laguna de Champaxan (Altamira, Tamaulipas, Mexico) and for *P. formosa* in 1993 from the Rio Purification (Barretal, Tamaulipas, Mexico)*,* respectively. The fish were sacrificed on ice and the gonads were quickly excised, pooled into one sample for each species and frozen in liquid nitrogen. With regard to animal welfare, we followed internationally recognized guidelines and applicable national legislation. We received ethical approval from the deputy of animal welfare of the University of Potsdam.

The frozen tissues were moved to 600 μl RLT buffer (Qiagen), 6 μl β-mercaptoethanol (48.7%; Promega), and glass beads (0.75 - 1.0 mm, BioSpec Products) soaked in RNase away (Thermo Scientific). After the homogenization of the samples with a bead beater, the total RNA was extracted with the RNeasy® Fibrous Tissue Mini Kit (Qiagen) following the manufacturer’s protocol. Quality and concentration of the isolated RNA were measured with a NanoDrop (ND-1000, Thermo Scientific) spectrophotometer. A MINT-Universal cDNA synthesis kit (Protocol I, Evrogen) was used for construction of the cDNA and subsequently the cDNA was purified with a NucleoSpin® Gel and PCR Clean-up kit (Machery-Nagel) according to the manufacturer’s protocols.

Sequencing of both non-normalized cDNA libraries as single read libraries (100 bp) was performed on one lane of an Illumina HiSeq 2000 sequencing system by a commercial provider (LGC Genomics GmbH, Berlin). This company also provided initial processing of the raw sequencing data with the analysis pipeline Casava (v1.8, Illumina Inc.) and the software FastQC (v0.9.2, Babraham Bioinformatics). Through quality reporting with the control tool FastQC, most of the sequencing errors appearing in any Illumina sequencing run (especially at the 3′-end) could be removed from the dataset, including the removal of all reads containing an unknown base character (‘N’), the trimming of the reads at the 3′ end to obtain reads with an average Phred quality score of at least 20 over a ten base window and the removal of reads shorter than 35 bp after trimming.

### ***De novo*** transcriptome assembly and detection of contamination

The assembly of the transcriptome was accomplished using the software packages Velvet (v1.2.03) [24] and Oases (v0.2.06) [25], which are suitable to assemble a transcriptome with short reads in absence of a reference genome (*de novo*). For the assembly of both transcriptomes, the following settings were used: minimum k = 61, maximum k = 69, k steps = 4.

To detect potential contamination, all contigs were compared with protein sequence databases from different taxa using the software BLAST (v2.2.26+) [26]. The protein sequences for archaea, bacteria, fungi, and invertebrates were downloaded (May 2012) from the universal protein knowledgebase UniProtKB (Swiss-Prot & TrEMBL) which contains manually annotated entries (UniProtKB/Swiss-Prot) and furthermore entries which are computationally annotated (UniProtKB/TrEMBL) [27]. The content of each database was reduced by removing redundant sequences with 95% identity using the software CD-Hit (v4.5.4) [28]. All contigs of both species were compared with the clustered databases via the blastx algorithm, which translates the query sequences in all six possible frames. Contigs that had a match were subsequently blasted against a protein database of the zebrafish, *Danio rerio,* downloaded from NCBI (July 2012) [29]. The cut-off for each taxon was defined according to jumps within the distribution of the E-values. Transcripts with hits above the taxon-specific E-value cut-off were identified as contamination and excluded from further analyses.

### Transcriptome annotation and comparative analysis

A challenge in genomics and transcriptomics investigations is to make the large amount of data accessible for further - mainly bioinformatic – analysis. A mandatory step is to annotate the data, i.e., to provide information about the biological background of the sequences based on the nucleotide, protein, and process level [30]. Sequences were therefore assigned to gene ontology (GO) terms that describe the biological process, cellular components, and molecular functions associated with a given gene product. The annotation of the transcriptomes was done with the software package GOblet (standalone local installation, November 2012) [31,32]. GOblet utilizes sequence similarities to already annotated and characterized proteins of other species based on BLAST comparisons and annotates the sequences with terms from the Gene Ontology project [33]. The assembled transcripts of the Amazon molly and the Atlantic molly were compared via BLAST against UniProt/Swiss-Prot protein databases of vertebrates, rodents, mammals, human, and invertebrates (November 2012) using only records with GO-annotations which are not inferred from electronical annotation (IEA) and an E-value cut-off of 10^−10^ was chosen.

The occurrences of the 148 generic GO terms (http://www.geneontology.org/GO_slims/goslim_generic.obo, February 2013) within the annotated data were computed and for each of the GO terms a Fisher’s exact test (α = 0.05) with false discovery rate (FDR) correction of the p-value was carried out to detect GO terms, which are over- or underrepresented in one of the two transcriptomes.

Furthermore, both transcriptomes were compared with protein and cDNA datasets downloaded from NCBI (March 2012) of *Danio rerio* (zebrafish), *Oryzias latipes* (medaka)*, Oreochromis niloticus* (nile tilapia), the NCBI Unigene entries of *Gasterosteus aceleatus* (three-spined stickleback), and the Uniprot/Swiss-Prot (March 2012) database. The sequence similarity comparisons against the protein databases were conducted with the blastx algorithm. The tblastx algorithm used for the cDNA databases translates the query and furthermore the database nucleotide sequences in all six possible frames. For the NCBI databases the E-value cut-off was 10^−50^ and for the UniProt/Swiss-Prot 10^−5^. The best hits for each contig were scanned for specific expressions within the meiosis and reproduction related GO terms and already described genes involved in meiosis [34-37] and thus detected genes were afterwards tested for the occurrence of open reading frames (ORFs) on the OrfPredictor server [38] as well as the complete transcript data for each species.

Additional to the mentioned sequence similarity comparisons with the different databases the assembled transcripts were also compared with the available datasets of the transcriptomes of Atlantic molly specimens from southern Mexico [unique loci in the assembled transcriptome, [39] and the guppy, *Poecilia reticulata*, a congeneric species [assembled 454 contigs, [40].

## Results

### Transcriptome sequencing

The sequencing of the cDNA libraries generated 117,702,546 reads for the Amazon molly and 114,683,463 for the Atlantic molly (Table 1). Through quality control and trimming, the raw dataset was reduced to 83,504,382 (70.95%) reads with 7,854,663,223 bp for *P. formosa* and 81,625,840 (71.17%) reads with 7,698,453,781 bp for *P. mexicana* and the average length of the reads was 94 bp for both species. The error probability that a base was incorrectly called in a sequence read is specified by the Phred quality. For the datasets of both species, the average Phred value was about 35, corresponding to an error probability of 0.00035.

**Table 1:** Transcriptome sequencing results (100 bp, single end)

**Species**	***Poecilia formosa***	***Poecilia mexicana***
Reads (n)	117,702,546	114,683,463
Filtered reads (n)	89,267,205	87,221,251
Trimmed reads (n)	83,504,382	81,625,840
Total bases (bp)^T^	7,854,663,223	7,698,453,781
Average read length^T^	94	94
Average Phred quality^T^	34.5	34.6

T: After trimming.

The processed reads for both species are available at the Sequence Read Archive (SRA, http://www.ncbi.nlm.nih.gov/sra) with the ID number PRJNA200586 for the Amazon molly and PRJNA200587 for the Atlantic molly.

### Assembly and contamination load

**Figure 1:**
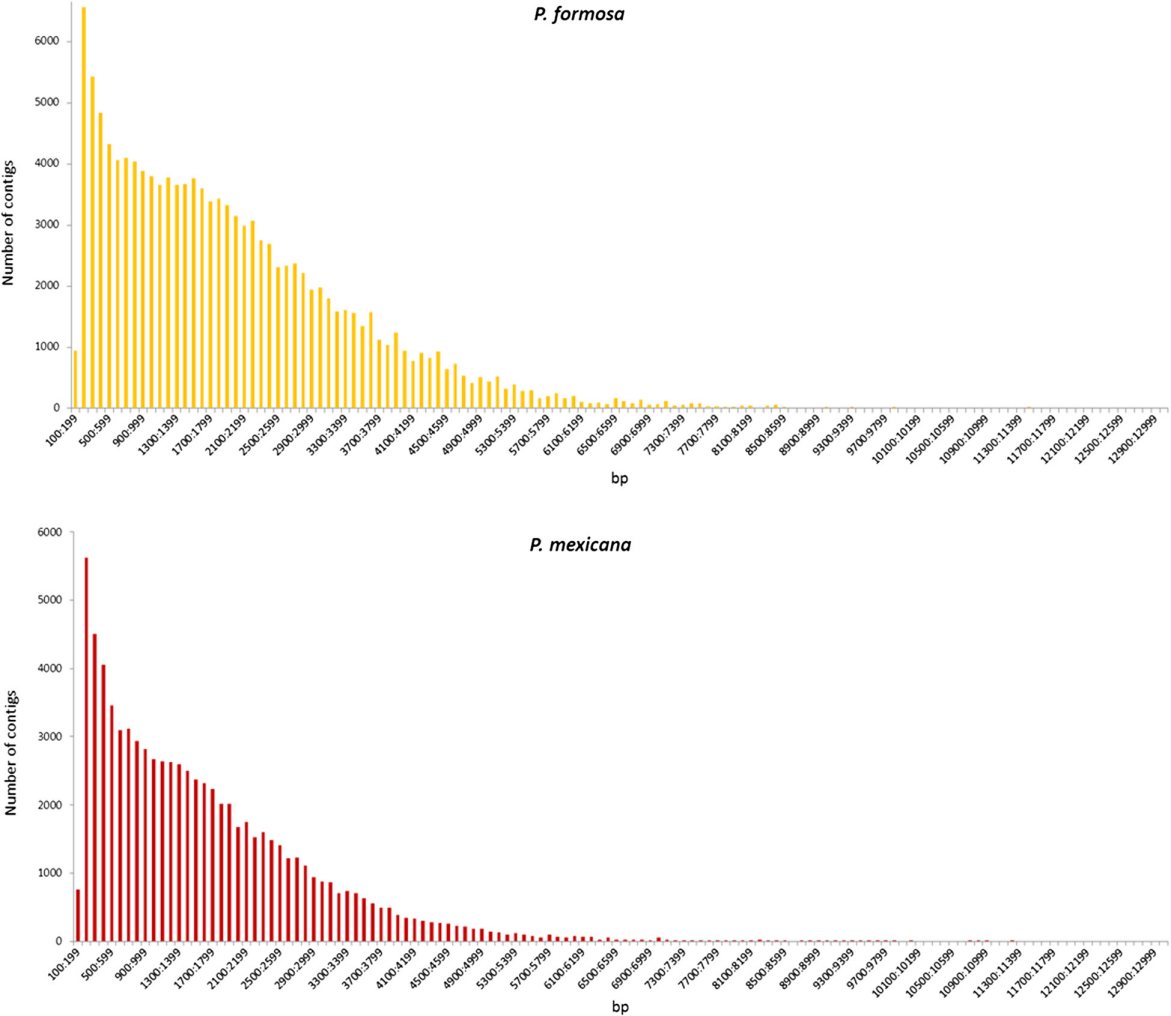
**Length distribution of the contigs for the Amazon molly (*P. formosa*) and for the Atlantic molly (*P. mexicana*).**

The *de novo* assembly with the Velvet/Oases software package yielded 127,283 contigs for the Amazon molly and 78,961 contigs for the Atlantic molly (Table 2) with an average length of 1,990 bp (range: 102 – 13,078 bp) and 1,638 bp (range: 100–11,242 bp), respectively. The N50 value reflects the quality of an assembly and is described by the weighted median of the contigs, which was 2,809 bp for *P. formosa* and 2,363 bp for *P. mexicana*. The length distribution of the assembled contigs for both species is shown in Figure 1.

**Table 2:** **Statistics for the *de novo *assembly**

**Species**	***Poecilia formosa***	***Poecilia mexicana***
Total bases (bp)	253,322,491	129,352,105
Total contigs* (n)	125,991	78,223
N50 (bp)	2,809	2,363
GC Content (%)	46.63	45.60

*Without contigs presumably originating from contamination (see text for details).

The sequence similarity comparisons with the UniProt/Swiss-Prot databases identified 1,291 contigs (1.02%) for the Amazon molly and 738 contigs (0.93%) for the Atlantic molly that were likely contaminations. These contigs were therefore removed from the transcriptome datasets.

### Transcriptome annotation and identification of candidate genes

**Figure 2:**
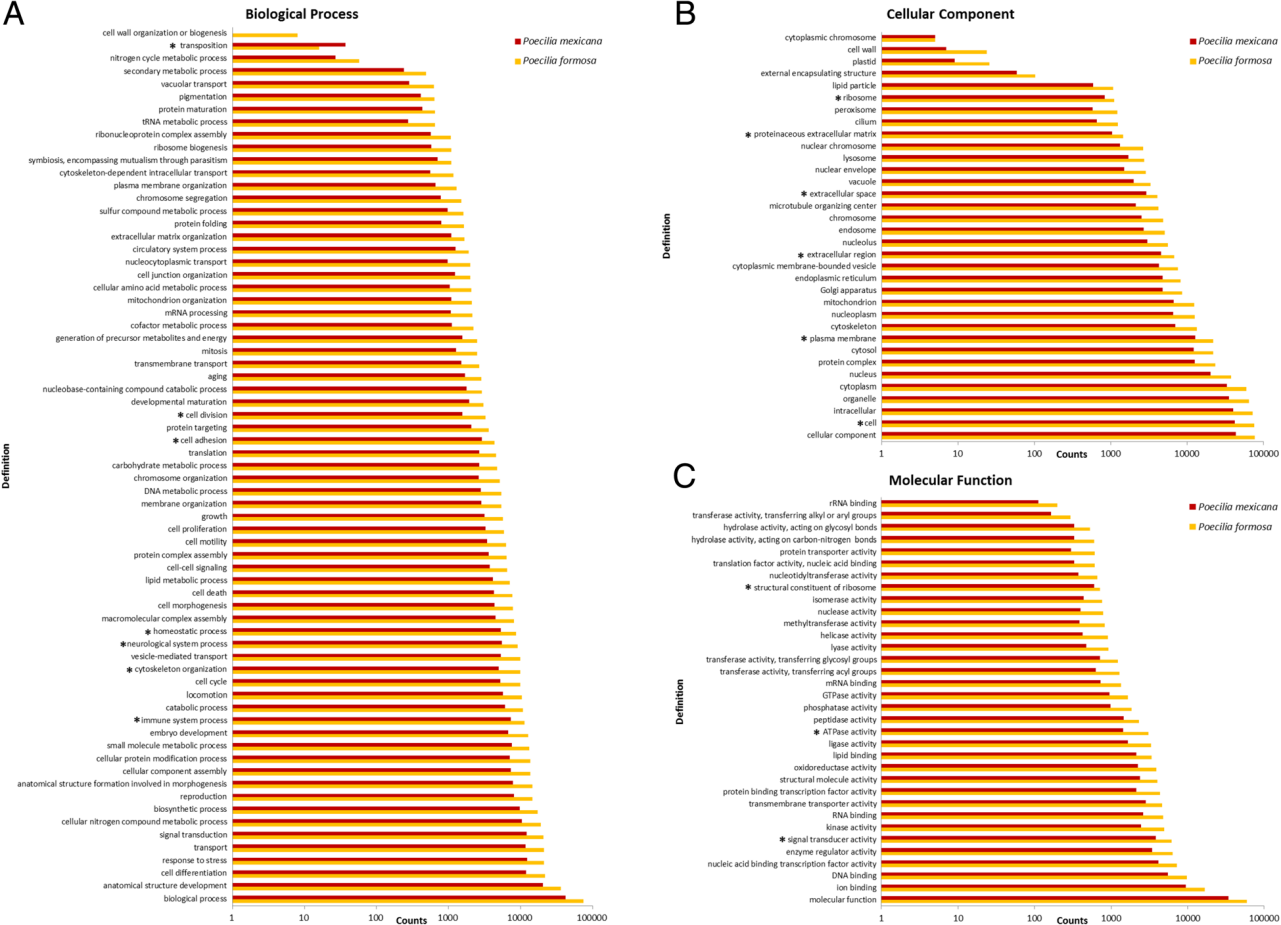
**Occurrence of the represented GO terms within the annotated transcriptomes of the Amazon molly (*P. formosa*) and the Atlantic molly (*P. mexicana*).** The classification of the GO terms are shown for the main categories “Biological process” **(A)**, “Cellular component” **(B)** and “Molecular function” **(C)**. Significant differences between the two species are labelled with an asterisk.

79,656 contigs (63.23%) for the Amazon molly and 44,785 (57.26%) for the Atlantic molly were assigned to a total of 12,328 different GO terms. 331 of these were unique to *P. formosa* (673 contigs) and 341 to *P. mexicana* (430 contigs). From the 148 generic GOslim terms 137 occurred within each transcriptome. The distribution of the GO term counts was very similar in both species, but 16 GO terms showed significant differences between the two species (Figure 2).

In comparisons with genomic resources of different taxa, the lowest number of hits was obtained with the three-spined stickleback (Table 3). The BLAST searches against the cDNA or protein databases of the zebrafish gave similar results for the Amazon molly and the Atlantic molly. This also applies for the comparisons with the databases of the medaka and the nile tilapia. Also the comparison of our data with published transcriptomes of the genus *Poecilia*[39,40] gave approximately equivalent results for *P. formosa* and *P. mexicana* (Table 4). The number of unique matches for the UniProt/Swiss-Prot database was 14,537 (16.10%) for *P. formosa* and 13,749 (26.53%) for *P. mexicana*. Of these matches, 10,929 were shared across the two species, while 3,608 (3.99%) and 2,820 (5.44%) hits were unique for the Amazon molly and the Atlantic molly, respectively. The best hits for each contig of these BLAST searches were screened for genes involved in reproduction, recombination, and especially in meiosis. In total, 75 such genes could be detected within the transcriptomes of the Amazon molly and Atlantic molly (Table 5), corresponding to 940 contigs, i.e., 630 contigs (67.02%) for the Amazon molly and 310 contigs (32.98%) for the Atlantic molly. All these contigs have open reading frames. The subset of the 75 genes contained seven genes only found in *P. formosa* (Ago4, Cdk14, Cdk16, Rad1, Smarca2, Smarca4, Xrcc3), while six were only found in *P. mexicana* (C15orf60, Mcm4, Zmcm3, Msh5, Rad9B, Rmi2). Smarca2 and Smarca4 - both only found in the transcriptome of the Amazon molly, represented by 11 and 10 contigs, respectively - are members of the SWI/SNF protein family, which are involved in transcription regulation [41,42].

**Table 3:** Summary of BLAST comparisons

**Taxa**		**Entries**	**BLAST algorithm**	***Poecilia formosa***	***Poecilia mexicana***
*Gasterosteus aculeatus*	cDNA	16,728	tblastx	45,098 (35.79%)	24,653 (31.51%)
*Danio rerio*	cDNA	28,037	tblastx	72,993 (57.94%)	39,005 (49.86%)
	Protein	27,014	blastx	72,045 (57.18%)	38,368 (49.05%)
*Oryzias latipes*	cDNA	22,456	tblastx	78,151 (62.03%)	42,575 (54.43%)
	Protein	22,078	blastx	76,539 (60.75%)	41,422 (52.95%)
*Oreochromis niloticus*	cDNA	23,339	tblastx	78,231 (62.09%)	42,999 (54.97%)
	Protein	23,162	blastx	76,301 (60.56%)	41,744 (53.34%)
Swiss-Prot	Protein	534,695	blastx	90,288 (71.66%)	51,821 (66.25%)

Shown are for each taxon the number of the sequences of the cDNA and protein databases, the BLAST algorithm used, and the number of hits (percentage) for the Amazon molly (*P. formosa*) and the Atlantic molly (*P. mexicana*).

**Table 4:** **Comparison to published transcriptomes of the genus *Poecilia***

**Taxa**	**Entries**	***Poecilia formosa***	***Poecilia mexicana***
*Poecilia mexicana*[39]	53,245	24,635 (46.27%)	24,574 (46.15%)
*Poecilia reticulata*[40]	54,987	11,843 (21.54%)	12,039 (21.89%)

Listed are the number of hits (percentage) of the available cDNA entries of the genus *Poecilia* compared through the tblastx algorithm (E-value cut-off was 10^−50^) with our Amazon molly (*P. formosa*) and Atlantic molly (*P. mexicana*) transcriptomes.

**Table 5:** **Identified genes, their UniProt accession IDs and the number of corresponding contigs for the Amazon molly (*P. formosa; Pofo*) and the Atlantic molly (*P. mexicana; Pome*)**

**Gene name**	**Description**	**UniProt accession ID**	**Number of contigs**	
			**Pofo**	**Pome**
Ago1	Argonaute 1, Eukaryotic translation initiation factor 2C 1	Q8CJG1	1	7
Ago2	Argonaute 2, Eukaryotic translation initiation factor 2C 2	Q8CJG0	10	2
Ago3	Argonaute 3, Eukaryotic translation initiation factor 2C 3	Q9H9G7	8	9
Ago4	Argonaute 4, Eukaryotic translation initiation factor 2C 4	Q9HCK5	9	0
C15orf60	Meiotic recombination protein REC114-like	Q7Z4M0	0	2
CcnA1	Cyclin-A1	Q92161	13	7
CcnA2	Cyclin-A2	P30274	20	8
CcnC	Cyclin-C	Q28F72	8	3
Cdk1	Cell division protein kinase/Cyclin-dependent kinase 1	Q9DG98	3	3
Cdk2	Cell division protein kinase/Cyclin-dependent kinase 2	P43450	1	1
Cdk4	Cyclin-dependent kinase 4	Q91727	9	4
Cdk7	Cyclin-dependent kinase 7	P51953	18	2
Cdk10	Cell division protein kinase/Cyclin-dependent kinase 10	Q2TBL8	1	1
Cdk14	Cell division protein kinase/Cyclin-dependent kinase 14	B0VXL7	3	0
Cdk16	Cell division protein kinase/Cyclin-dependent kinase 16	Q00536	3	0
Mcm2	DNA helicase MCM2, Minichromosome maintenance protein 2	Q6DIH3	10	1
Mcm3	DNA helicase MCM3, Minichromosome maintenance protein 3	Q5ZMN2	1	3
Mcm4	DNA helicase MCM4, Minichromosome maintenance protein 4	P33991	0	2
Mcm4B	DNA replication licensing factor MCM4-B, Minichromosome maintenance protein 4-B	P30664	3	3
Mcm5	DNA helicase MCM5, Minichromosome maintenance 5	Q561P5	18	2
Mcm6	DNA helicase MCM6, Minichromosome maintenance 6	Q14566	3	4
Mcm7	DNA helicase MCM7, Minichromosome maintenance 7	Q6NX31	18	3
Mcm8	DNA helicase MCM8, Minichromosome maintenance 8	Q9UJA3	15	2
Mcm9	DNA helicase MCM9, Minichromosome maintenance 9	Q6NRM6	19	1
Zmcm3	Zygotic minichromosome maintenance protein 3	Q7ZXZ0	0	4
Mei1*	Meiosis inhibitor protein 1	Q5TIA1	11	11
Mlh1*	DNA mismatch repair protein Mlh1, MutL protein homolog 1	P40692	7	4
Mlh3*	DNA mismatch repair protein Mlh3, MutL protein homolog 3	Q9UHC1	6	1
Mnd1*	Meiotic nuclear division protein 1 homolog	Q32L19	11	3
Mns1*	Meiosis-specific nuclear structural protein 1	Q6PBA8	1	2
Mre11	Double-strand break repair protein MRE11	Q9W6K1	2	1
Msh2*	DNA mismatch repair protein Msh2, MutS protein homolog 2	Q5XXB5	10	6
Msh3*	DNA mismatch repair protein Msh3, MutS protein homolog 3	P20585	8	4
Msh4*	DNA mismatch repair protein Msh4, MutS protein homolog 4	O15457	7	4
Msh5*	DNA mismatch repair protein Msh5, MutS protein homolog 5	O43196	0	4
Msh6*	DNA mismatch repair protein Msh6, MutS protein homolog 6	P52701	26	6
Piwil1	Piwi-like protein 1	Q8UVX0	13	6
Piwil2	Piwi-like protein 2	A6P7L8	7	2
Pms2	DNA mismatch repair protein (endonuclease) PMS2	P54278	12	1
Psmc3ip*	Homologous-pairing protein 2 homolog (HOP2)	Q63ZL2	3	2
Rad1	Cell cycle checkpoint protein RAD1	Q5R7X9	4	0
Rad21*	Double-strand-break repair protein rad21 homolog	O60216	9	12
Rad50	DNA repair protein RAD50	P70388	2	2
Rad51B*	DNA repair protein RAD51 homolog B	Q91917	23	3
Rad51C*	DNA repair protein RAD51 homolog 3	O43502	7	1
Rad51D*	DNA repair protein RAD51 homolog 4/D	O75771	7	5
Rad52	DNA repair protein RAD52 homolog	P39022	10	11
Rad54A*	DNA repair and recombination protein RAD54-like	Q92698	18	6
Rad54B*	DNA repair and recombination protein RAD54B	Q9DG67	9	1
Rad9A	Cell cycle checkpoint control protein RAD9A	Q99638	8	3
Rad9B	Cell cycle checkpoint control protein RAD9B	Q6WBX8	0	1
Rec8*	Meiotic recombination protein REC8 homolog	O95072	3	2
RecQl1	ATP-dependent DNA helicase Q1	Q9Z129	3	3
RecQl4	ATP-dependent DNA helicase Q4	O94761	3	6
RecQl5	ATP-dependent DNA helicase Q5	O94762	5	2
Rmi1	RecQ-mediated genome instability protein 1	A4IF98	15	1
Rmi2	RecQ-mediated genome instability protein 2	Q5ZM20	0	2
Sfr1*	Swi5-dependent recombination DNA repair protein 1 homolog, Meiosis protein 5 homolog	B7ZD04	2	1
Smarca2	SWI/SNF-related matrix-associated actin-dependent regulator of chromatin subfamily A member 2	Q6DIC0	11	0
Smarca4	SWI/SNF-related matrix-associated actin-dependent regulator of chromatin subfamily A member 4	A7Z019	10	0
Smc1a*	Structural maintenance of chromosomes protein 1A	Q9CU62	10	2
Smc1b*	Structural maintenance of chromosomes protein 1B	Q8NDV3	2	2
Smc2*	Structural maintenance of chromosomes protein 2	P50533	9	2
Smc3*	Structural maintenance of chromosomes protein 3	Q9CW03	2	3
Smc4*	Structural maintenance of chromosomes protein 4	P50532	7	8
Smc5*	Structural maintenance of chromosomes protein 5	Q802R9	4	1
Smc6*	Structural maintenance of chromosomes protein 6	Q6P9I7	24	3
Smchd1	Structural maintenance of chromosomes flexible hinge domain-containing protein 1	A6NHR9	25	37
Spo11*	Meiotic recombination protein	Q9Y5K1	4	11
Stag1*	Cohesin subunit SA-1	Q8WVM7	3	13
Stag2*	Cohesin subunit SA-2	Q8N3U4	7	14
Xrcc1	DNA repair protein XRCC1	Q60596	21	3
Xrcc2*	DNA repair protein XRCC2	Q9CX47	19	12
Xrcc3*	DNA repair protein XRCC3	Q08DH8	2	0
Xrcc4	DNA repair protein XRCC4	Q924T3	24	6

*Genes that are specific for meiosis [34-37].

Among the 75 genes, 31 genes could be identified which are supposedly specific to meiosis. One example is the meiosis-specific Spo11 gene, which encodes for the SPO11 protein, a type II topoisomerase-like enzyme that is essential for the formation of double-strand breaks (DSBs) between homologous chromosomes during meiosis [43,44]. Four contigs of the Amazon molly and eleven of the Atlantic molly matched to the Spo11 gene.

For each species, one unique meiosis-specific gene was detected. The Xrcc3 gene codes for a protein involved in DNA damage repair [45] and was only found in the transcriptome of the Amazon molly. The Msh5 gene, found only in the transcriptome of the Atlantic molly, is a eukaryotic homolog of the mutS genes and the protein MSH5 participates as heteroduplex together with the MSH4 protein in meiotic recombination [46,47].

## Discussion

This study is the first description of the transcriptome of an unisexual vertebrate. Such organisms are models for studying the evolution and maintenance of sexual recombination. Most importantly, we use a comparative approach to study the gynogenetic Amazon molly, *P. formosa* relative to its maternal ancestor the Atlantic molly, *P. mexicana*. Our obtained datasets add to a small, yet growing, number of genomic data available for the paradigmatic subfamily Poeciliinae (phylogeny according to [48]) and provides relevant information for different future research topics.

About 115 million reads for each sample were obtained by Illumina sequencing of two unnormalized cDNA libraries on one lane. After trimming and quality control, ~70% (15.5 Gb) of the sequence reads were used for assembly and annotation.

A transcriptome of *P. mexicana*[39] and of the guppy, *P. reticulata*[40], have already been described, but neither the genome of the Amazon molly nor the genomes of the two ancestor species are currently available. So far, only the genome of another member of the subfamily Poeciliinae, the platyfish (*Xiphophorus maculatus*) has been studied extensively [49]. Consequently, the assembly of the reads was conducted without a reference genome (*de novo*), and therefore the sequencing coverage had to be higher than 30x [50]. In our study, the coverage was 52.11x for the Amazon molly and 57.89x for the Atlantic molly.

The unequal number of contigs for the Amazon molly and the Atlantic molly can be ascribed to a higher number of transcripts predicted for the Amazon molly during assembly, leading to a different number of base pairs that were utilized for assembly by the assembly software (~250 million bp for the Amazon molly and ~130 million bp for the Atlantic molly). This difference can be explained by the fact that the Amazon molly is a hybrid species and therefore has two different alleles at any locus, i.e., one from the maternal ancestor, *P. mexicana* and one of the paternal ancestor, *P. latipinna*. In all other aspects, i.e., coverage, average read length of the assembled contigs, and theN50 value, both species were quite similar. In addition, the contamination load of about 1% for each contig set was very low. Most of the contigs presumably originating from contamination were assigned to invertebrates, but some contaminations could be assigned to fungi and bacteria. Such contamination can occur at different steps of the library preparation or the sequencing.

The results of the BLAST searches against the different fish species showed similar results for both species. The percentage of database matches did not correlate with phylogenetic relationship to the mollies, but rather with the completeness of the genomic resources. The smaller number of matches with the three-spined stickleback is due to the fact that this database is smaller and presumably less complete than those of the other fish model species. The medaka, *O. latipes,* is the more closely related species to both molly species [51], but most matches were found with the more distantly related zebrafish, *D. rerio,* which is a well established model organism and thus has a well investigated and annotated genome and the highest number of entries in the database. The comparisons among transcriptomes within the genus *Poecilia* reveal a congruence between number of shared transcripts and phylogenetic relationship: As expected, the number of hits of our new assembled transcriptomes was higher to the unique loci of *P. mexicana*[39] than to those of *P. reticulata*[40]. The high concordance of both transcriptomes with the well-established UniProt/Swiss-Prot database was used for the identification of candidate genes. 75 genes associated with reproduction and meiosis were detected within the contig sets for both species. 31 genes of these are specific for meiosis, like the Spo11 genes or the Msh genes. Nonetheless, several genes that also play a central role during meiosis are not listed in our table of the candidate genes. One example is the Dmc1 gene, which is a member of the SMC protein family. The DMC1 protein is required for meiotic recombination, especially for the homologous pairing of chromosomes during meiosis [52]. In fact, we detected this gene within the transcriptomes of both species, but with a too high E-Value to consider the assignment reliable.

The identification of meiosis-specific genes within the transcriptome of the Amazon molly raises questions concerning the function of these genes and their encoded proteins, as the unisexual Amazon molly reproduces via gynogenesis, and the diploid eggs are produced by apomixis i.e., without meiotic reduction [19]. The meiosis-specific genes have thus lost their putative prime purpose. Their function – if any - in the gonads of an apomictic species remains enigmatic. It would be interesting to evaluate the abundance of the respective proteins, both in gonads and other tissue. This could provide some hint as to whether these genes and proteins maintain their original function or shifted to other functions. A first step would be to examine the tissue-specific expression patterns of these genes across various types of tissue via real-time polymerase chain reaction.

The differential occurrence of some meiosis-specific genes between the Amazon molly and the Atlantic molly as well as the differences in contig numbers can - as above mentioned – be interpreted by the hybrid nature of the Amazon molly. To corroborate this hypothesis, we will compare the transcriptomes of all three species, i.e., the Amazon molly and both parental species.

## Conclusions

Here we describe the first transcriptome analysis of the all-female and hybrid species *P. formosa*, the Amazon molly, and the first transcriptome of any unisexual vertebrate of hybrid origin. The transcriptome was assembled and annotated without a reference genome, using short single end reads obtained through Illumina sequencing. Through comparisons with the transcriptome of the maternal ancestor *P. mexicana* and BLAST searches against other fish species and the UniProt database, a significant number of candidate genes relevant for reproduction and especially meiosis could be identified. The obtained dataset, and especially the identified meiosis-specific genes can act as starting point for further studies like gene-/tissue – specific expressions analysis. The transcriptomic data of the second ancestor species, *P. latipinna*, and a well annotated genome of the Amazon molly – when becoming available – will allow for a more comprehensive understanding of the genetic architecture of the Amazon molly.

## Competing interests

The authors declare that they have no competing interests.

## Authors’ contributions

IMS conducted the lab work, the bioinformatic analysis and drafted the manuscript. SH and DG contributed to the bioinformatics. IS and RT conceived the study, supervised the work, and contributed to the manuscript. All authors read and approved the manuscript.
